# Use of Community Science Data to Assess Factors Associated With Diamond‐Backed Terrapin (*Malaclemys terrapin*) Sightings in Coastal Marshes of Southeastern North Carolina, USA

**DOI:** 10.1002/ece3.71526

**Published:** 2025-06-17

**Authors:** Morgan Whitmer, Elizabeth Pinnix, Sarah Finn, Jessie Jarvis, Hope Sutton, Amanda Southwood Williard

**Affiliations:** ^1^ University of North Carolina Wilmington Wilmington North Carolina USA; ^2^ North Carolina Wildlife Resources Commission Raleigh North Carolina USA; ^3^ North Carolina Coastal Reserve Wilmington North Carolina USA

## Abstract

The diamond‐backed terrapin ( *Malaclemys terrapin* Schoepff, 1793) is an elusive species that is difficult to study due to its semi‐aquatic nature and a lack of specific information about where populations occur. The aim of this study was to assess the factors associated with diamond‐backed terrapin sightings at two estuarine monitoring sites in North Carolina using data collected via community science head count surveys. Incorporating community scientists into long‐term ecological studies allows researchers to increase the scope of data collection while engaging community members in conservation efforts for species of concern. Diamond‐backed terrapin sighting data were used as the response variable in binomial models that included time of day, air temperature, habitat type, season, and distance from the nearest possible nesting location as explanatory variables. Diamond‐backed terrapins were significantly more likely to be sighted during morning surveys and during Spring compared with Fall at both study sites. There was a negative correlation between air temperature and diamond‐backed terrapin sightings. At one of the two study sites, there was a positive correlation between distance from the nearest possible nesting beach and diamond‐backed terrapin sightings, and diamond‐backed terrapins were significantly more likely to be sighted in aquatic habitats with mud substrate. Spatial and temporal patterns in diamond‐backed terrapin sightings may be influenced by foraging patterns and behaviors associated with thermal and osmotic regulation, as well as environmental conditions that affect detectability. The results of this study lay the groundwork for additional studies of diamond‐backed terrapin presence and abundance at our study sites, expansion of survey efforts into different regions, and broader efforts to assess population responses to environmental and anthropogenic stressors in this salt marsh species.

## Graphical abstract

The aim of this study was to assess the factors associated with diamond‐backed terrapin sightings at two estuarine monitoring sites in North Carolina using data collected via community science head count surveys. Spatial and temporal patterns in diamond‐backed terrapin sightings may be influenced by foraging patterns and behaviors associated with thermal and osmotic regulation, as well as environmental conditions that affect detectability. The results of this study lay the groundwork for additional studies of diamond‐backed terrapin presence and abundance at our study sites, expansion of survey efforts into different regions, and broader efforts to assess population responses to environmental and anthropogenic stressors in this salt marsh species. Photo credit – Jared Oviatt.

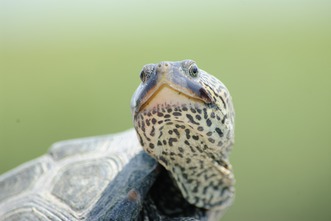

## 1 Introduction

The diamond‐backed terrapin ( *Malaclemys terrapin* Schoepff, 1793) is the only obligate estuarine turtle in the western hemisphere, inhabiting salt marshes, tidal flats, and lagoons along the Atlantic and Gulf Coasts of North America (Coker 1906; Morris et al. 2011; Denton et al. 2016; Agha et al. 2018). This semi‐aquatic species utilizes aquatic habitats for foraging and mating, and terrestrial sites for basking, overwintering, and nesting (Harden et al. 2007). The diamond‐backed terrapin is unique among North American turtles in its preference for aquatic habitats with high and variable salinity, and co‐occurrence with other turtle species is rare, with the exception of marine turtles (Ernst and Lovich 2009). Diamond‐backed terrapins exhibit distinct seasonal patterns in habitat use, with overwintering mud burial in supratidal and intertidal zones and a higher percentage of time spent in aquatic habitats during the warmer months. From spring through fall, diamond‐backed terrapins take advantage of flooded salt marsh cordgrass ( *Spartina alterniflora* Loisel, 1807) stands to forage on a variety of invertebrate prey (e.g., mollusks, crustaceans, and annelids; Coker 1906; Bishop 1983; Tucker et al. 1995; Spivey 1998; McKee et al. 2016) and utilize intertidal muddy habitats for basking or mud burial as a means of thermoregulation or osmoregulation during low tide (Davenport and Macedo 1990; Davenport and Magill 1996; Harden et al. 2007; Williard and Harden 2011; Akins et al. 2014). This species nests in sparsely vegetated, sandy terrestrial habitats just above the intertidal zone during the spring and early summer (Roosenburg 1994; Szerlag‐Egger and McRobert 2007; Cook et al. 2018).

Diamond‐backed terrapins were subject to direct fisheries harvest to meet consumer demands for turtle soup in the early 20th century (Roosenburg 1994; Roosenburg and Green 2000; Rook et al. 2010; Converse et al. 2017), and steep population declines were documented during this time (Coker 1906; Hildebrand 1929; Walker and Jones 2018). Direct fishing pressure on diamond‐backed terrapin populations eased as the commercial demand for turtle soup declined (Gibbons et al. 2001); however, quantitative data on terrapin population trends over the subsequent decades are lacking. Although diamond‐backed terrapins are no longer a target species for fisheries, they currently face a variety of threats in both their terrestrial and aquatic habitats, including habitat alteration and loss of habitat due to coastal development (Isdell et al. 2015; Maerz et al. 2018), road mortality (Crawford et al. 2014), mortality due to human‐subsidized predators (Feinberg and Burke 2003; Draud et al. 2004), and mortality due to incidental capture in commercial and recreational crab pots (Dorcas et al. 2007; Chambers and Maerz 2018). There is a general consensus among scientists and management agencies that diamond‐backed terrapin populations are declining region‐wide (Bishop 1983; Roosenburg et al. 1997, 2019; Gibbons et al. 2001; Dorcas et al. 2007), and local extirpations have been documented at study sites in South Carolina for which long‐term data are available (Gibbons et al. 2001; Tucker et al. 2001; Dorcas et al. 2007). Consequently, the diamond‐backed terrapin is listed as Vulnerable by the International Union for Conservation of Nature (IUCN) Red List (Roosenburg et al. 2019) and, although currently not listed as a federally protected species under the United States Endangered Species Act, the species is afforded protection under state agency regulations throughout its range.

In the state of North Carolina, the diamond‐backed terrapin is listed as a “Species of Special Concern” by the North Carolina Wildlife Resources Commission (NCWRC), and directed collection and/or possession of the species is restricted. The semi‐aquatic nature of the diamond‐backed terrapin and use of remote and/or concealed habitats for various aspects of life history present challenges for population monitoring. To date, field studies and observations of diamond‐backed terrapins in North Carolina have been focused on a limited number of study sites (Hart and Crowder 2011; Harden and Willard 2012). Expansion of surveys to identify additional areas in which diamond‐backed terrapins occur is necessary to provide a comprehensive statewide assessment of population status and trends. Both direct capture and observational methods may be used to document diamond‐backed terrapin presence in a given habitat. Previous studies have utilized seine nets, trammel nets, bank traps, fyke nets, gill nets, and modified crab traps to capture and mark individual diamond‐backed terrapins for long‐term population monitoring (Lovich and Gibbons 1990; Tucker et al. 2001; Roosenburg et al. 1997; Dorcas et al. 2007; Selman et al. 2019). These methods are time‐consuming, labor‐intensive, and require prior knowledge of population locations (Harden et al. 2009). Recent studies have adopted visual head count surveys as a less intensive method to identify areas utilized by diamond‐backed terrapins and, in some cases, generate abundance estimates (Harden et al. 2009; Lovich et al. 2018; Breininger et al. 2019; Levasseur et al. 2019). This observational method may be expanded to include community scientists, thus providing opportunities to increase the geographic scope and frequency of data collection (Altwegg and Nichols 2019; MacPhail and Colla 2020). Studies that utilize community scientists have the capacity to track wildlife populations over extended periods of time and evaluate historical changes in the context of environmental conditions (MacPhail and Colla 2020). Well‐designed programs that foster collaborations between scientists and community members have contributed significantly to concrete conservation outcomes for a wide range of threatened species (Fontaine et al. 2022). Community engagement in large‐scale data collection for species monitoring provides the additional benefits of public awareness, involvement in local conservation efforts, and promotion of scientific approaches (McKinley et al. 2017; Altwegg and Nichols 2019).

The NCWRC, in collaboration with the North Carolina Coastal Reserve & National Estuarine Research Reserve (NCCR), initiated a community science diamond‐backed terrapin head count survey (i.e., Terrapin Tally) in 2014 to document diamond‐backed terrapin sightings at multiple sites along the southeastern coastline of the state. The kayak‐based head count surveys involve paddling a designated route and recording GPS coordinates of terrapin sightings using a smartphone application. Terrapin Tally surveys provide wildlife managers with a means of identifying areas with confirmed presence of diamond‐backed terrapins so that appropriate sites for long‐term monitoring may be identified. Expansion of these community science surveys into other coastal areas would enhance the capacity for data collection by management agencies, provide a more comprehensive assessment of diamond‐backed terrapin distribution, and identify additional sites for diamond‐backed terrapin research and population monitoring.

The goal of this study was to use data on diamond‐backed terrapin sightings collected by trained community science volunteers, along with environmental data collected by our research team, to generate models to assess the environmental and spatiotemporal factors associated with diamond‐backed terrapin sightings at established survey routes in southeastern North Carolina. The results of this study lay the groundwork for investigations of diamond‐backed terrapin presence and abundance at our study sites, expansion of survey efforts into different regions, and broader efforts to assess population responses to environmental and anthropogenic stressors in this salt marsh species. Our study also contributes to the growing body of evidence that supports the use of community science as a means to engage the public in local conservation efforts, promote the use of scientific approaches to conservation, and contribute to long‐term datasets for management of species of concern.

## 2 Materials and Methods

### 2.1 Study Sites

**FIGURE 1:**
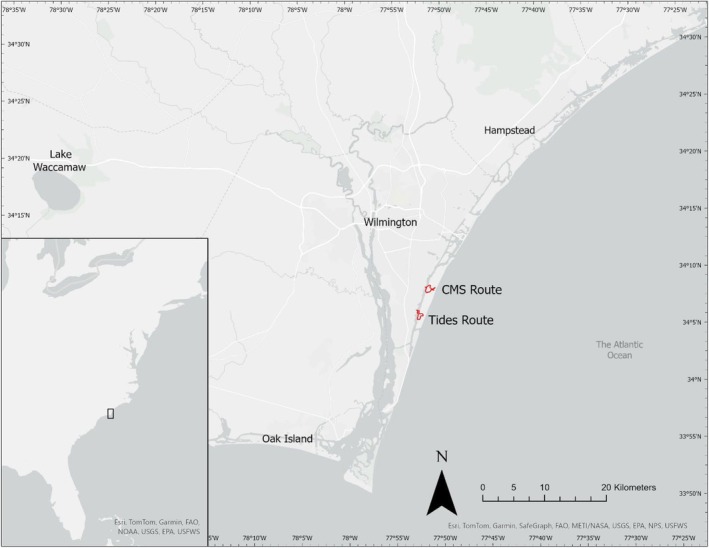
Map indicating the location of study site in Southeast North Carolina (34°07′19.2″ N, 77°51′17.4″ W). Kayak routes for terrapin surveys at Masonboro Island are indicated in red: CMS route starting point 34°14′04.3″ N, 77°86′35.1″ W, Tides route starting point 34°10′68.6″ N, 77°87′93.6″ W.

Research was conducted at the Masonboro Island Reserve in southeastern North Carolina, a 23 km^2^ preserve bounded by the Atlantic Ocean and the Intracoastal Waterway that is managed by the NCCR (Figure 1). The Masonboro Island Reserve encompasses a barrier island and the surrounding salt marsh, tidal creeks, and tidal flats with water salinity close to coastal ocean values (25–35 psu, Davis et al. 2019). The NCCR has mapped multiple survey routes along the channels and creeks adjacent to and within the Masonboro Island Reserve to be used for community science diamond‐backed terrapin head count surveys. For our study, we selected two frequently monitored NCCR survey routes along which diamond‐backed terrapins are regularly sighted: Center for Marine Science (CMS; route starting point at 34°14′04.3″ N, 77°86′35.1″ W) and Tides (route starting point at 34°10′68.6″ N, 77°87′93.6″ W) (Figure 1). The starting point for the two surveys is separated by a distance of 4.0 km.

### 2.2 Field Surveys

Over the course of the Spring (May–June), Summer (July—August), and Fall (September—October) of 2019, a total of 37 kayak‐based diamond‐backed terrapin head count surveys were performed by community scientists and University of North Carolina Wilmington (UNCW) student volunteers. All survey participants are required to complete an online orientation and an in‐person training session, during which they are provided with detailed information about diamond‐backed terrapin natural history and identification, survey protocols and field procedures, and water safety. Participants completed a practice survey prior to collecting data. The surveys were restricted to within 2 h of high tide, as many of the tidal creeks in the Masonboro Island Reserve are not navigable at mid‐ to low tide. Surveys were conducted between sunrise and sunset, with efforts made to distribute surveys equally across the morning and afternoon hours, and restricted to periods when winds were less than 16 kph (10 mph). Air temperature was documented at the beginning of each survey based on National Oceanographic and Atmospheric Administration weather buoy data (https://www.ndbc.noaa.gov, stations MBNN7 and NOXN7). Salinity at the survey routes varies with tidal cycle and weather conditions, and generally falls within the range of 25–35 psu (Davis et al. 2019). Given the survey protocol (i.e., continuous movement along the survey route, please see below) it was not feasible to record salinity at each diamond‐backed terrapin sighting location. Diamond‐backed terrapins are the only species of Emydid turtle routinely encountered in the tidally influenced barrier island habitats in which the community science surveys took place. While some other North American Emydid and Chelydrid turtles tolerate brackish water, no other species occurs at the consistently high salinities along our survey routes.

For each of the 37 surveys, a team of two surveyors was assigned to each route (CMS and Tides); both routes were paddled concurrently. Surveyors used a mobile phone map application (Avenza Maps, Avenza Systems Inc., Toronto, Canada) to track their position along the designated survey route and document diamond‐backed terrapin sightings. For each team of two surveyors, one surveyor was responsible for navigating the route, and the other surveyor was responsible for recording data from sightings. Both paddlers scanned the water and shoreline up to approximately 30 m from their kayaks in a semicircle of 180°. An observation of diamond‐backed terrapin head(s) seen at the surface of the water or along the banks of tidal creeks constituted a diamond‐backed terrapin sighting. Participants identified diamond‐backed terrapins based on characteristics of the head, including size and coloration. Diamond‐backed terrapin head size is considerably smaller than that of marine turtle species that may periodically enter the barrier island tidal creeks and bays, and diamond‐backed terrapin head coloration is highly distinct with white to gray skin speckled with black spots. Diamond‐backed terrapin sightings within ~30 m from the kayak were recorded using the Avenza application, which automatically documented the geographic coordinates of the observation. Observers were encouraged to paddle at a consistent speed and be highly vigilant. All kayak surveys were conducted in accordance with UNCW boater safety procedures. Upon completion of the surveys, data entered via the Avenza application was uploaded to a database for storage and analysis.

**FIGURE 2:**
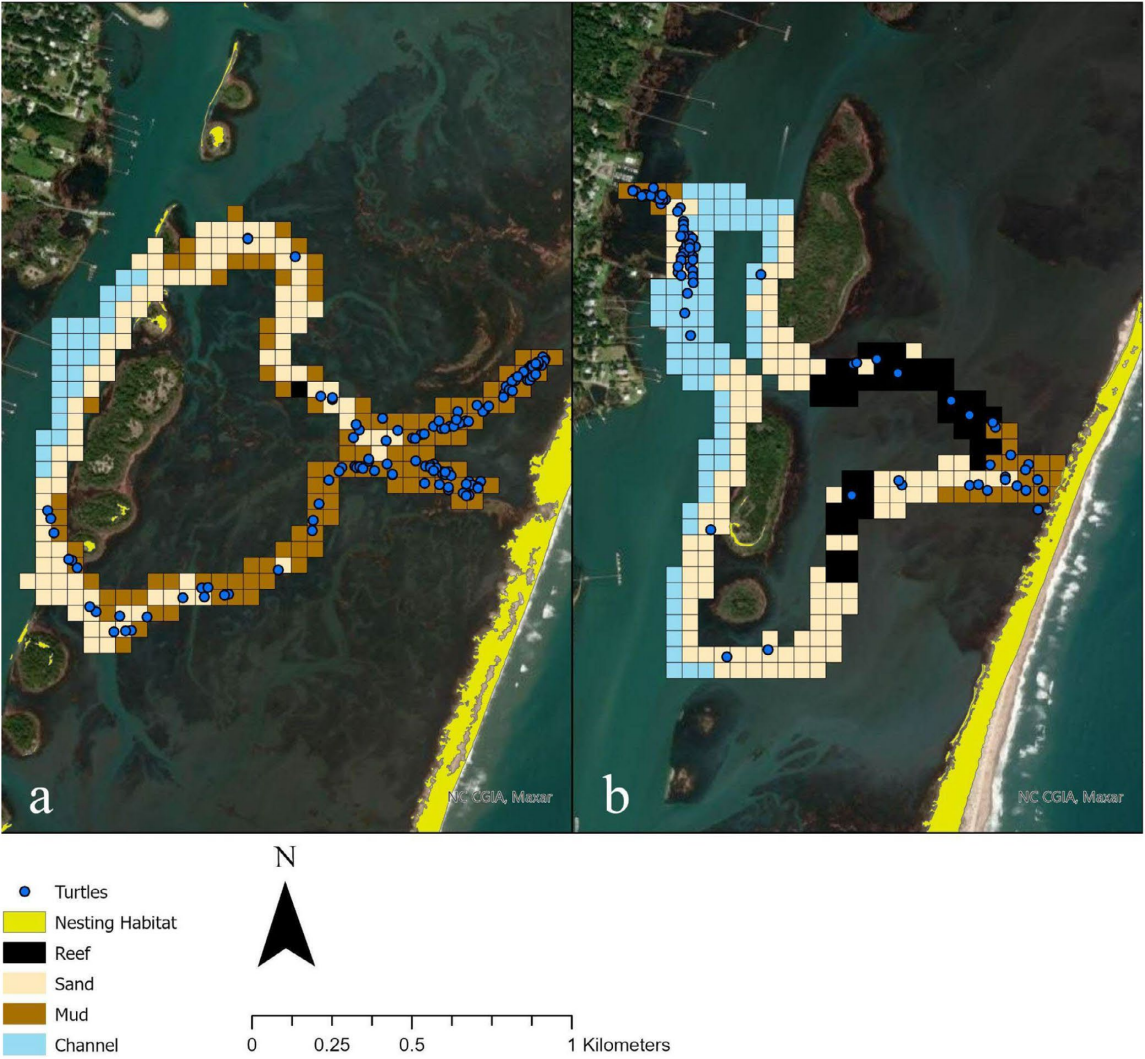
Diamond‐backed terrapin survey routes (a—CMS route, b—Tides route) within the Masonboro Island Reserve overlaid with 50 × 50 m grid cells assigned a color‐coded habitat type as indicated by the legend. The GPS coordinates for each diamond‐backed terrapin sighting are shown as dark blue circles.

Aerial drone surveys were conducted over the entirety of each route to provide imagery for data analysis in ArcGIS 10.6.1 (ESRI Redlands, California). Drone surveys were accomplished using a Phantom 3 Professional drone (DJI Developer Technologies Shenzhen, China). Images were taken at 400 ft. (121 m) per federal flight limitations. Drone surveys were conducted at low tide within two hours following first light to provide imagery of the underwater structure of the estuary for characterization of habitat type (see Section 2.3). Images taken from drone surveys were compiled into one mosaic image in Agisoft (Agisoft LLC St. Petersburg, Russia) and uploaded into ArcGIS 10.6.1. A tessellation tool was used to create a grid of 50 m × 50 m grid of cells along each route (Barnas et al. 2019), and each grid cell was assigned a number (1–482). The GPS coordinates for each terrapin sighting were overlaid on the grid; thus, each individual terrapin location was associated with a specific grid cell (Figure 2).

### 2.3 Habitat Assessment

Drone imagery was used to classify habitat type for each grid cell along the survey routes based on the National Estuarine Research Reserve System (NERRS) Habitat and Land Cover Classification Scheme (Garfield et al. 2008): Mud, Sand, Reef, and Channel (Table 1 and Figure 2). We conducted site visits to visually confirm habitat type and ensure that habitat identification using drone imagery was accurate. A random point with associated GPS coordinates was assigned within each grid cell; then a random number generator was used to select random points for field quadrat surveys. Field sampling was performed at ~10% of the random points. Fourteen points were sampled on the CMS route, and 19 points were sampled on the Tides route. Field sampling was conducted using a quadrat method with 1 m × 1 m, ½″ PVC EL 90D frames. A digital photograph of the estuary floor structure within the quadrat was taken at each randomly selected point during low tide to corroborate habitat determinations made using drone imagery. Once we confirmed that the habitat type identified in the quadrat matched our identification of habitat type based on drone imagery, we were confident in assigning habitat types along the entirety of each survey route using the drone imagery. Each grid cell along the survey routes was assigned one habitat type based on the predominant cover within the grid cell. The percent cover for each habitat type along each survey route was estimated using drone imagery.

**TABLE 1:** Habitat descriptions based on National Estuarine Research Reserve System (NERRS) criteria (Garfield et al. 2008).

Habitat	Description
Sand	Unconsolidated substrate particles smaller than stones are predominantly sand
Mud	Unconsolidated substrate particles smaller than stones are predominantly saturated or submerged silt and clay
Reef	Includes ridge‐like or mound‐like structures formed by the colonization and growth of sedentary invertebrates mixed substrate comprised of sand, silt and clay
Channel	Permanently Flooded: Water covers the land surface throughout the year in all years

Potential diamond‐backed terrapin nesting locations were identified using data collected during the NERRS habitat mapping effort conducted in 2010. Terrestrial areas within the Masonboro Island Reserve characterized by open (i.e., unvegetated) expanses of sandy soil that excluded dune were characterized as potential nesting sites for diamond‐backed terrapins. The distance from the center of each survey route grid cell to the closest possible nesting location was measured using the Measure tool in ArcGIS.

### 2.4 Statistical Analysis

Data were examined for outliers, collinearity, and variance inflation factors prior to analysis (Zuur et al. 2007). The effect of abiotic drivers on terrapin sightings was analyzed separately by survey route (CMS and Tides); the global model for both survey routes included time of day, air temperature, habitat type, season, and distance from the nearest possible nesting location as covariates. Following data exploration, the large number of zeros in the total number of observations resulted in the selection of a zero‐inflated binomial (ZIB) generalized linear model to describe detection (1) and non‐detection (0) data. The ZIB regression model for terrapin detection/non‐detection is given by:

(1) $${\text{Terrapins}}_{i}\sim \mathrm{ZIB}\left(\mu_{i},\pi \right)$$

where the detection/non‐detection portion of the model is the mean number of observations (both positive observations and zeros) at location i, and π is the probability of recording zeros only (Zuur et al. 2012). To relate the means of both these distributions of the model to a global linear mixed model, we used link functions defined as:

(2) $$\begin{array}{c} \log\left(\mu_{i}\right)=\beta_{1}+\beta_{2}\times {\text{Time}}_{i}+\beta_{3}\times \mathrm{air}\;{\text{temp}}_{i}\\ +\beta_{4}\times {\text{Habitat}}_{i}+\beta_{5}\times {\text{Season}}_{i}+\beta_{6}\times {\text{Distance}}_{i}+\varepsilon_{i} \end{array}$$

For both:

(3) $$\text{logit}\left(\pi \right)=\gamma$$

where $\beta_{x}$ are the regression parameters and $\varepsilon_{i}$ is the spatial random effect at location *i*. From this we calculated the expected value and variance of detection or non‐detection of terrapins at each location.

### 2.5 Bayesian Inference Using INLA

Integrated Nested Laplace approximations (INLA), a computationally efficient alternative to Markov chain Monte Carlo (MCMC) methods, were used to calculate the distribution of the regression parameters (Rue et al. 2009; Benguin et al. 2012) using the INLA package. We used two separate approaches to account for spatial autocorrelation and selected the approach that provided the best fit for the data: (1) we ran the INLA models with a spatial mesh, and (2) we ran the INLA models without the spatial mesh but incorporated grid cell as a random effect in the model. For the models with the spatial mesh, we specified the use of a Gaussian Markov Random Field (GMRF) to reduce computational time, and sampling locations were converted into areal triangulations using stochastic partial differential equations (SPDE) (Rue and Held 2005; Lindgren et al. 2011). The spatial correlation structure of the residuals was then calculated using the Matérn correlation function (Cressie 1993; Lindgren et al. 2011). All models assumed a GMRF prior for the intercept, regression parameters, and covariates with a mean of 0 and a sparse precision matrix.

After running the global model for each approach, all non‐relevant covariates were removed, and the model was re‐run until only relevant covariates remained (Zuur et al. 2007). For each model, residuals were also inspected for spatial correlation using variograms (Cressie 1993) in the gstat and sp packages (Pebesma 2004; Bivand et al. 2008). The best‐fit model was the simplest model with the lowest Watanabe‐Akaike information criteria (WAIC) (Gelman et al. 2014). The random effects structure (grid cell versus spatial mesh) was explicitly compared, and grid cell inclusion emerged as the superior model based on WAIC scores. All statistical analyses were conducted in the software environment R v.4.3.0 (R Core Team 2023).

## 3 Results

We documented a total of 180 diamond‐backed terrapin sightings over the course of the 37 surveys (Figure 2); a total of 32 community science volunteers participated in the surveys. The INLA models with the incorporation of grid cells as a random effect to account for spatial autocorrelation, rather than the spatial mesh, provided the best fit for the data at both survey routes. Table 2 provides the WAIC scores for the five top models at each survey route; Table 3 provides summary statistics of the INLA models of best fit for each survey route and indicates which effects were statistically significant based on 95% credible intervals (CI). There was a negative correlation between air temperature and terrapin sightings, and terrapins were significantly more likely to be sighted during morning surveys compared with afternoon surveys at both survey routes. There was no statistically significant difference in terrapin sightings between Spring and Summer, but significantly fewer terrapins were sighted in Fall compared with Spring at both survey routes. At the Tides survey route, terrapins were significantly more likely to be sighted in habitats with mud substrate, and there was a positive correlation between distance from the nearest possible nesting beach and terrapin sightings.

**TABLE 2:** Model comparison showing the Watanabe‐Akaike information criteria (WAIC) scores of the top five models for each survey route (CMS and Tides).

Model parameters	WAIC
CMS route	
Sighting ~ time + temp + season + f(grid)	1645.65
Sighting ~ time + temp + f(grid)	1652.79
Sighting ~ temp + season + f(grid)	1659.75
Sighting ~ temp + f(grid)	1667.63
Sighting ~ time + season + f(grid)	1680.02
Tides route	
Sighting ~ time + temp + habitat + season + distance + f(grid)	3865.40
Sighting ~ time + temp + habitat + distance + f(grid)	4368.25
Sighting ~ time + habitat + distance + f(grid)	4407.41
Sighting ~ habitat + season + distance + f(grid)	4860.18
Sighting ~ time + habitat + season + distance + f(grid)	4960.09

*Note:* The global model included time of day (time), air temperature (temp), habitat, season, and distance to the nearest potential nesting site (distance) as covariates; grid cell was included as a random effect to account for spatial autocorrelation.

**TABLE 3:** Summary statistics for INLA models of diamond‐backed terrapin sightings at the CMS and Tides survey routes in the Masonboro Island Reserve in southeastern North Carolina.

Fixed effect	Mean	SD	95% CI (lower, upper)
CMS route			
Intercept^a^	−2.464	0.93	−4.281, −0.629
Time – PM^a^	−0.739	0.179	−1.096, −0.395
Air temperature^a^	−0.093	0.032	−0.032, −0.093
Season – Summer	−0.005	0.197	−0.395, 0.378
Season – Fall^a^	−0.775	0.228	−1.231, −0.336
Tides route			
Intercept^a^	4.933	1.458	2.158, 7.841
Time – PM^a^	−1.186	0.249	−1.683, −0.704
Air temperature^a^	−0.296	0.046	−0.389, −0.206
Season – Summer	0.097	0.258	−0.404, 0.609
Season – Fall^a^	−1.85	0.338	−2.519, −1.190
Habitat – Sand^a^	−1.432	0.317	−2.095, −0.865
Habitat – Reef^a^	−1.926	0.389	−2.721, −1.196
Habitat – Channel^a^	−3.368	0.455	−4.312, −2.539
Distance from potential nest site^a^	0.0034	0	0.002, 0.004

*Note:* The comparison group was Spring for the categorical fixed effect of Season, and the comparison group was Mud for the categorical fixed effect of Habitat. Posterior mean, posterior standard deviation (SD), and 95% credible intervals (CI) are provided for each fixed effect in the model of best fit.

^a^ Statistically significant based on exclusion of Zero from the 95% CI.

## 4 Discussion

Establishing whether a species is threatened with extinction and warrants protection requires sufficient data on population status, trends, demography, and threats. Effective population monitoring may be limited by the logistics of field sampling for species of concern, such as the diamond‐backed terrapin, that exhibit complex life history patterns, uneven distribution, elusive behaviors, and/or the use of habitats that are remote or challenging for humans to access (Thompson 2004). There are few long‐term population monitoring sites for diamond‐backed terrapins, but the limited data that exist indicate there has been a decrease in population numbers over the past 30 years, as well as shifts in demography that could affect reproductive success (Gibbons et al. 2001; Dorcas et al. 2007; Roosenburg et al. 2019). Expansion of monitoring to encompass additional sites throughout the diamond‐backed terrapin's range is necessary for a comprehensive assessment of the overall population status and trends over time, as well as the impacts of specific threats on diamond‐backed terrapin populations. Field surveys to document diamond‐backed terrapin sightings are time‐consuming and labor‐intensive. Our study utilized data collected by community science volunteers to conduct rapid assessments to document diamond‐backed terrapin sightings. Community science surveys can serve as a useful first step to identify target areas for additional studies and the establishment of long‐term monitoring sites to estimate diamond‐backed terrapin abundance and evaluate population trends. Identification of spatiotemporal features and environmental conditions associated with the detection of diamond‐backed terrapins may be used to guide more effective survey efforts and refine strategies for species management.

Diamond‐backed terrapins utilize a variety of tidally influenced habitats, including shallow bays, tidal creeks, mangroves, and seagrass meadows, across a broad geographic range (Brennessel 2006). The presence of diamond‐backed terrapins in a given area may be governed by site‐specific ecological and environmental factors. Results from our study indicate that habitat substrate is a predictor of terrapin sightings. Diamond‐backed terrapins occurred in all four habitat types along the Tides survey route, but diamond‐backed terrapin sightings were significantly higher in aquatic habitats with mud substrate compared with sand substrate, reef, or deeper channels along this route. Habitat type was not included in the model of best fit for the CMS survey route, but this may reflect the difficulty of modeling zero‐inflated data rather than the relative importance of substrate type in diamond‐backed terrapin habitat utilization at this study site. Of the 115 diamond‐backed terrapin observations at the CMS survey route, 96 (83.5%) were in habitats with mud substrate, 19 (16.5%) were in habitats with sand substrate, and no diamond‐backed terrapin observations were made in reef or channel habitats (Figure 2). The low number of diamond‐backed terrapin observations overall, combined with the lack of observations in two of the four habitat types, could not be accommodated by the ZIB regression model we used to assess detection/non‐detection data for the CMS survey route.

Utilization of mud substrate habitats may reflect the foraging preferences of diamond‐backed terrapins and their use of mud burial as a component of overwintering and osmoregulatory strategies (Davenport and Magill 1996; Harden et al. 2014). Diamond‐backed terrapin diet varies with ontogeny and body size but typically includes species of gastropods (e.g., *Littorina irrorata* Say, 1822, *Melampus bidentatus* Say, 1822) and crustaceans (e.g., *Callinectes sapidus* Rathbun, 1896, *Uca pugnax* Smith, 1870) that are commonly found in mud substrate habitats (Tucker et al. 1995; Spivey 1998). Marsh periwinkle snails ( *Littorina irrorata* Say, 1822) are notably abundant in the salt marshes surrounding Masonboro Island survey routes and likely serve as a food source for diamond‐backed terrapins (Tucker et al. 1995). In addition to the use of mud substrate habitats for foraging, diamond‐backed terrapins in southeastern North Carolina use exposed muddy habitats in the intertidal zone for basking and mud burial throughout the year. Harden and Willard (2012) found that diamond‐backed terrapins spend up to 20% of their time during the summer and 87% of their time during the winter buried in the mud substrate of the intertidal zone. Mud burial may reduce passive exchange of water and salts between the animal and the environment and permit maintenance of osmotic homeostasis, especially during periods of cold exposure when metabolic means of osmoregulation are limited (Williard et al. 2019).

Diamond‐backed terrapins emerge from overwintering mud burial in the Spring and aggregate in shallow, sheltered locations where water temperatures are relatively warm (Butler et al. 2018). Previous studies have noted that the predicted abundance of diamond‐backed terrapins in waters near suitable nesting sites followed a hill‐shaped response during the Spring, with low abundance early in the season, peak abundance in mid‐June, and declining abundance through the Summer and Fall (Levasseur et al. 2022). We found a positive correlation between terrapin sightings and distance from the nearest potential nesting sites at the Tides survey route, which may reflect the timing of our surveys in relation to peak nesting activity for diamond‐backed terrapins; in other words, our observations may have occurred prior to or after the brief peak nesting period when diamond‐backed terrapins were most abundant near nesting sites. It should be noted that the location of nesting activities may be dynamic and influenced by weather events and coastal processes, such as erosion. Documentation of diamond‐backed terrapin nesting in the Masonboro Island Reserve is opportunistic, and there are no dedicated surveys to systematically locate and monitor nests on this remote barrier island. Consequently, our evaluation of proximity to nesting sites as a predictor of diamond‐backed terrapin sightings along in‐water survey routes is hampered by the lack of confirmed nesting sites and limited information about the timing of nesting activity. Diamond‐backed terrapin nesting beaches are typically narrow, sparsely vegetated sandy areas above the intertidal zone (Roosenburg 1994; Roosenburg and Kelley 1996; Mitchell and Walls 2013; Butler et al. 2018). Levasseur et al. (2022) found a positive correlation between the proximity of salt marsh habitat and diamond‐backed terrapin nesting habitat. The shallow‐water vegetated zones adjacent to nesting beaches may serve as foraging and staging areas for nesting females and provide protective habitat for post‐hatchlings (Selman 2018; Levasseur et al. 2022). Our surveys were limited to navigable channels during high tide, so we may not have been able to observe diamond‐backed terrapins that had aggregated in shallow‐water emergent vegetation directly adjacent to nesting beaches.

The ability of community science volunteers to observe diamond‐backed terrapins during surveys depends on the availability of the animal for detection and whether the animal is perceptible to the observer. Semi‐aquatic diamond‐backed terrapins along our survey routes are available to be sighted when they are at the water surface along the survey route; we would not be able to detect animals that were submerged (i.e., diving) or that were not in open water (i.e., buried in mud or concealed in marsh vegetation) (Sadykova et al. 2017; Breininger et al. 2019; Stolen et al. 2024). Furthermore, observer experience, habitat characteristics (e.g., broad channel or narrow creek), and environmental variables (e.g., glare and wind speed) may affect the ability of an observer to sight diamond‐backed terrapins that are present along the survey route (Levasseur et al. 2019, 2022). We found that diamond‐backed terrapins were more likely to be sighted in the morning compared with afternoon, which may reflect the effect of wind on water surface waves and turbidity and, consequently, the ability of observers to sight diamond‐backed terrapins at the water surface. Wind speeds are typically lower during the morning, and this may increase the ability of observers to detect diamond‐backed terrapins, particularly in less sheltered water bodies (Levasseur et al. 2019). An increased likelihood of sightings in the morning may also reflect differences in diamond‐backed terrapin activity patterns, rather than abiotic factors such as wind speed.

The semi‐aquatic nature of the diamond‐backed terrapin presents challenges for detection at all stages of the tidal cycle and during all seasons. Surveys conducted at high tide may fail to detect diamond‐backed terrapins that are foraging within flooded vegetation in the high marsh. On the other hand, surveys conducted at low tide may fail to detect diamond‐backed terrapins buried in the mud or basking on the mud surface in the intertidal zone. The effect of the tidal cycle on diamond‐backed terrapin sightings may vary depending on site‐specific characteristics and survey methods, and conflicting trends have been reported in the literature. Butler (2002) and Harden et al. (2009) sighted more diamond‐backed terrapins at low tide compared with high tide and speculated that this may reflect diamond‐backed terrapins retreating to deeper water creeks as high marsh foraging habitats become inaccessible with receding tides. In contrast, Levasseur et al. (2022) noted that diamond‐backed terrapin detections were highest at high tide and attributed this to an increase in foraging movements that brought diamond‐backed terrapins to areas that were more accessible to researchers. Our surveys were restricted logistically to high tide, as many of the creeks at Masonboro Island are not navigable at low tide. Diamond‐backed terrapins are likely to be foraging during high tide (Brennessel 2006; Levasseur et al. 2022), but only those individuals that were in the open water along our survey routes would be available for detection by observers. Nevertheless, results from our study are valuable for assessing areas with relatively high numbers of diamond‐backed terrapin sightings that may be good candidates for more intensive monitoring for studies of habitat utilization and population trends.

Diamond‐backed terrapin sightings were higher during the Spring compared with Fall, an observation that is in agreement with previously published results for head count surveys (Butler 2002; Harden et al. 2009; Levasseur et al. 2019, 2022). Seasonal shifts in aquatic and terrestrial habitat use (e.g., mud burial) and temperature‐induced changes in submergence patterns may contribute to seasonal differences in sightings. We found a negative correlation between air temperature and diamond‐backed terrapin sightings. Air temperatures during the course of our study varied between 16.3°C–34.1°C, within the range for diamond‐backed terrapin activity (Williard and Harden 2011). The trend toward an increase in sightings at lower air temperatures may reflect aquatic basking behavior of diamond‐backed terrapins. Akins et al. (2014) noted a higher incidence of aquatic surface basking in the Spring as terrapins absorb solar radiation to offset the loss of heat through conduction in cooler waters. Longer and more frequent periods of aquatic surface basking may result in higher diamond‐backed terrapin detectability and sightings in the Spring. Diamond‐backed terrapins may require less time at the water surface to meet thermoregulatory needs as both air and water temperatures increase over the course of the Spring and Summer. In contrast with our findings, Levasseur et al. (2019) found a positive relationship between diamond‐backed terrapin sightings and air temperature during shore‐based head count surveys. A potential explanation for this discrepancy is the cooler range of temperatures (11.1°C–32.2°C) over which Levasseur et al. (2019) observed diamond‐backed terrapins compared with our study. Diamond‐backed terrapins greatly reduce activity and exhibit terrestrial mud burial behaviors at cooler temperatures (Williard and Harden 2011; Akins et al. 2014), which could reduce observer ability to detect diamond‐backed terrapins that are present.

We used air temperature instead of water temperature in our models, as water temperature was variable with depth along survey routes and it was not logistically feasible for us to take water temperature measurements for each diamond‐backed terrapin sighting due to the survey protocol (i.e., continuous movement along the survey route). Other studies have documented a significant positive relationship between water temperature and diamond‐backed terrapin sightings (Butler 2002; Harden et al. 2009), which may reflect the effect of temperature on metabolic rate and dive times. Metabolic rate and oxygen consumption of ectothermic diamond‐backed terrapins increase with temperature (Williard and Harden 2011; Williard et al. 2019); consequently, dive times would be expected to be shorter, and diamond‐backed terrapins would be expected to surface more frequently as water temperatures increase. Dive times are not well studied in terrapins but may vary with season and temperature, as observed for other aquatic turtle species (Ultsch 2006). Additional studies to investigate dive cycle characteristics under various thermal conditions are necessary in order to improve our understanding of diamond‐backed terrapin availability for sighting during surveys and implications for assessing abundance and distribution.

Previous diamond‐backed terrapin head count surveys have used a study design in which observers conduct surveys from static points, either from shore or from a boat at specific locations within coastal waterbodies, in areas where terrapins previously have been documented (Isdell et al. 2015; Breininger et al. 2019; Levasseur et al. 2019; Stolen et al. 2024). In cases where distinct sampling sites are separated by distances greater than the typical home range of diamond‐backed terrapins, spatial autocorrelation is minimized and single‐species occupancy models or N‐mixture models can be used to estimate detection probability and abundance in relation to habitat features. In contrast, our survey involved continual, steady movement of the observers along a predesignated survey route. The benefit of this design is that it provides a rapid assessment tool to document diamond‐backed terrapin sightings over a relatively broad area and at novel coastal locations; however, this approach has limitations in terms of detection and spatial autocorrelation of observations, which need to be accounted for during analysis (Cressie 1993). In general, studies that utilized static point observations found a low detection probability for diamond‐backed terrapins (0.28–0.75; Isdell et al. 2015, Breininger et al. 2019); it is reasonable to assume that detection probabilities at our sites may be similar or perhaps even lower given the survey protocol of continual movement along the survey route. Low detection probability as well as low numbers of diamond‐backed terrapins at a given site contribute to zero‐inflated observation data. We used ZIB mixed effects models and the INLA Bayesian inference framework to account for zero‐inflated observation data and spatial autocorrelation (Sadykova et al. 2017). The INLA models do not directly incorporate detection probability, so our results do not provide information on the true abundance of diamond‐backed terrapins at a given site. That said, detection probability increases with an increase in species numbers in a given area (Altwegg and Nichols 2019) and previous diamond‐backed terrapin studies have noted that detection is highest in areas of high abundance (Breininger et al. 2019), so our survey protocol is likely to be effective in identifying areas of relatively high diamond‐backed terrapin abundance on which to focus additional conservation and management efforts. Recent advances in occupancy modeling allow users to account for spatial autocorrelation and imperfect detection in large data sets for single‐species models and integrated models that use multiple data sources (Doser et al. 2022). These approaches show promise for analyzing and interpreting data collected by community science volunteers and researchers.

Our survey approach and analysis provide information about the environmental variables and habitat characteristics associated with terrapin sightings, which may be used to inform future survey site selection and expansion of diamond‐backed terrapin monitoring. We found an association between diamond‐backed terrapin sightings and mud substrate habitats; this information may be useful in identifying novel sites for rapid assessment head count surveys within extensive estuarine systems where diamond‐backed terrapins may be present. The significant effect of season on diamond‐backed terrapin sightings should be considered when designing and implementing community science surveys. Given that sightings tend to decrease over the course of the active season due to ecological, behavioral, and physiological adjustments (Levasseur et al. 2019, 2022), implementing rapid assessment community science surveys during the Spring would maximize chances of observing diamond‐backed terrapins if they are present in a given area.

The utilization of community members in large‐scale data collection for species monitoring is widely beneficial for a variety of reasons, including enhancement of public awareness, involvement in local conservation efforts, promotion of scientific approaches, and contributions to long‐term datasets (McKinley et al. 2017; Altwegg and Nichols 2019). Confirmation of species occurrence within a given area is the first step toward detailed assessments of habitat use and population trends (Conroy et al. 2008), and our study illustrates the utility of community science head count surveys as a means to document diamond‐backed terrapin sightings. Our results lay the groundwork for additional studies of diamond‐backed terrapin presence and abundance at our study site, expansion of diamond‐backed terrapin survey efforts into different regions, and conservation actions to protect diamond‐backed terrapin populations. In the case of the Terrapin Tally community science project, data collected by community volunteers and regional researchers contributed to the development of management strategies to protect diamond‐backed terrapins from incidental capture and drowning in crab pots. Our Masonboro Island Reserve study site was designated as a Diamond‐backed Terrapin Management Areas (DTMAs) by the North Carolina Division of Marine Fisheries (NCDMF) in 2020 based on documented populations of diamond‐backed terrapins and the presence of habitat features associated with diamond‐backed terrapin bycatch vulnerability (Harden and Willard 2012; NCDMF 2020). Bycatch reduction technologies proven to significantly reduce the incidental capture of diamond‐backed terrapins are required on all commercial and recreational crab pots set within the boundaries of the DTMA. Expansion of DTMAs as a tool to protect diamond‐backed terrapins from bycatch mortality depends on knowledge of the spatial distribution and relative abundance of the species, but information about diamond‐backed terrapin occurrence along the North Carolina coastline is mostly limited to the few areas where directed research has been conducted (Spivey 1998; Hart and Crowder 2011; Harden and Willard 2012). The Terrapin Tally community science project, sponsored by the NCWRC and NCCR, initiated annual Spring surveys at the Masonboro Island Reserve in 2014 and has since expanded to include additional NCCR‐managed coastal reserves and other partner‐managed areas in the central and southernmost portions of the state. Use of community science head count surveys as a means of rapidly assessing locations where diamond‐backed terrapins occur and identifying areas for additional studies and long‐term monitoring may help guide efforts to implement DTMAs and other protective measures for this unique estuarine species.

## Author Contributions

**Morgan Whitmer:** conceptualization (equal), writing – original draft (lead), writing – review and editing (equal). **Elizabeth Pinnix:** conceptualization (equal), writing – review and editing (equal). **Sarah Finn:** conceptualization (equal), writing – review and editing (equal). **Jessie Jarvis:** formal analysis (lead), writing – review and editing (equal). **Hope Sutton:** conceptualization (equal), writing – review and editing (equal). **Amanda Southwood Williard:** conceptualization (equal), writing – review and editing (equal).

## Conflicts of Interest

The authors declare no conflicts of interest.

## Supporting information


Appendix S1.

Appendix S2.

Appendix S3.

## Data Availability

The data that supports the findings of this study are available in the Appendices [Link], [Link], [Link].
